# Supplementary material for: Motion-Based Extrinsic Sensor-to-Sensor Calibration: Effect of Reference Frame Selection for New and Existing Methods
Source: Sensors (Basel). 2023 Apr 4;23(7):3740. doi: 10.3390/s23073740 (PMC10098754; doi:10.3390/s23073740)
Supplement: Supplementary file 1 [file sensors-23-03740-s001.zip › sensors-2288421-supplementary.pdf]

Supplementary Materials: Motion-Based Extrinsic Sensor-to-Sensor Calibration: Effect of Reference Frame Selection for New and Existing Methods

Tuomas Välimäki *, Bharath Garigipati and Reza Ghabcheloo

1. Simulation experiments

Figures S1 to S5 present the calibration errors for all tested reference selection methods.

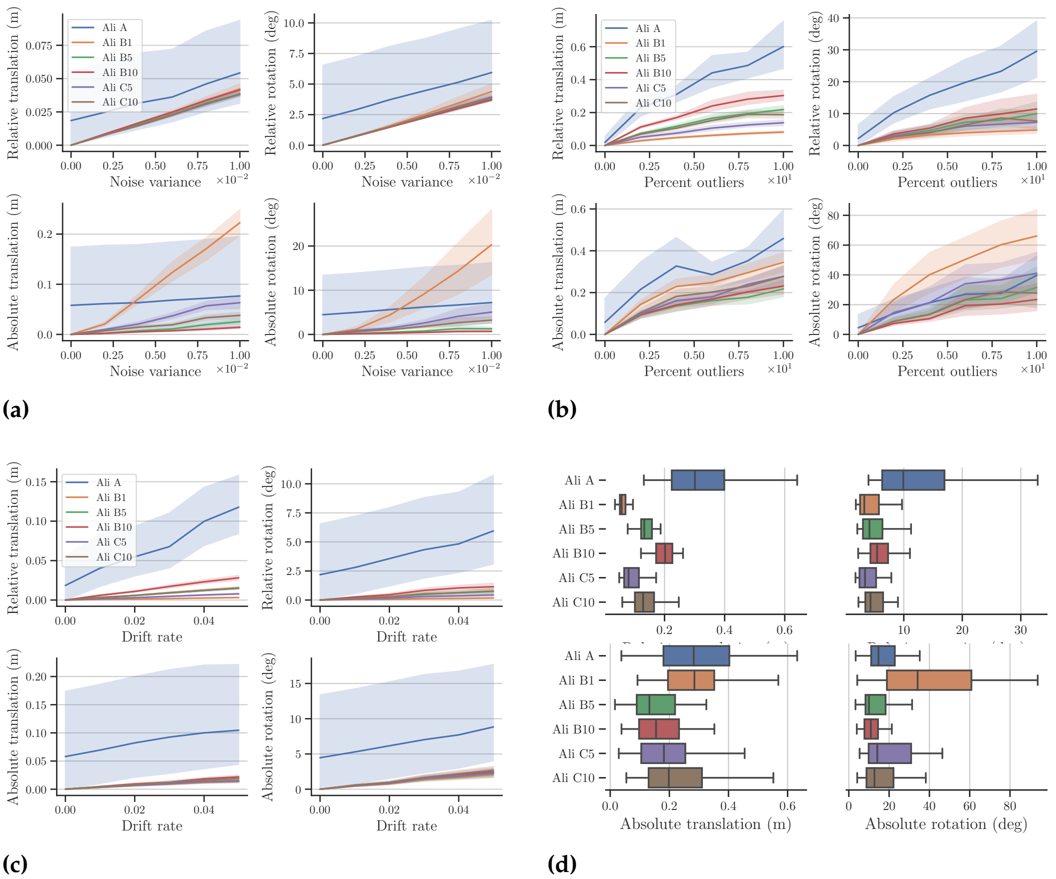

Figure S1. Calibration errors for Ali [1] on simulation data for all reference selection methods: (**a**) added Gaussian noise, (**b**) added outliers, (**c**) added drift, and (**d**) mixed noise. Plots (**a**) through (**c**) display the mean and 95% confidence intervals, whereas the boxplot (**d**) shows the median and quartiles.

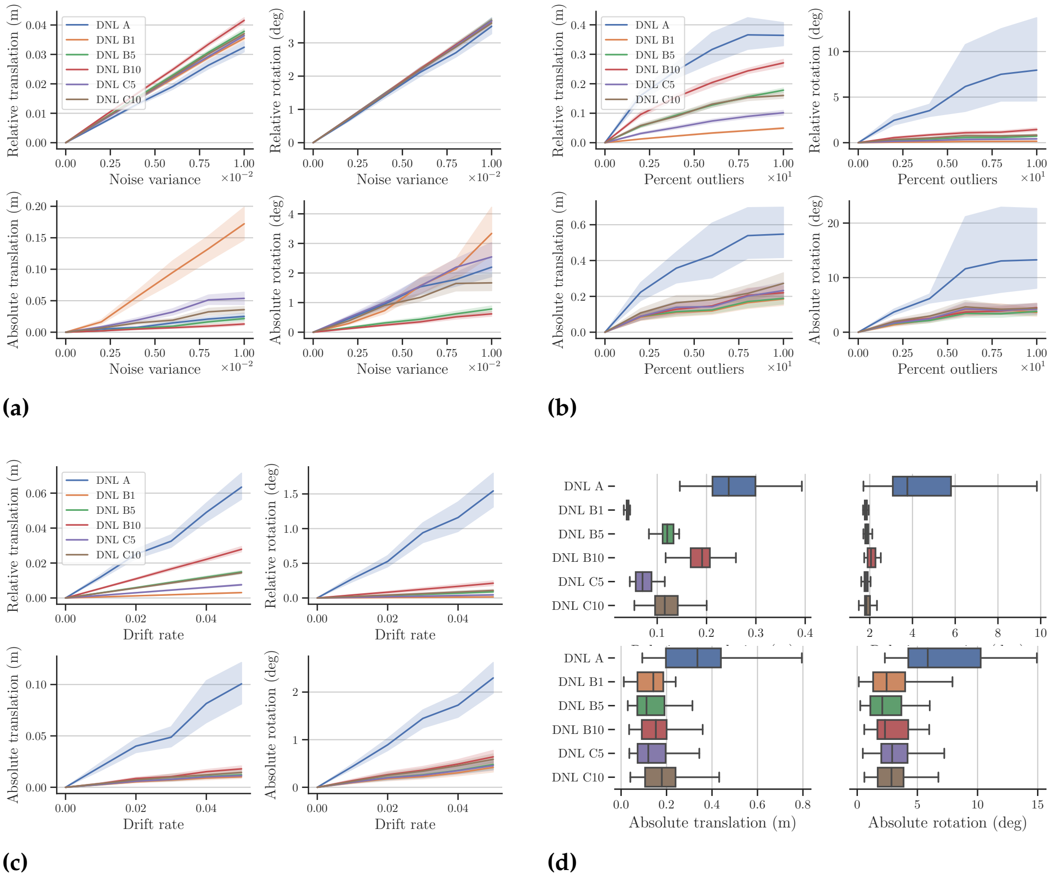

Figure S2. Calibration errors for DNL on simulation data for all reference selection methods: (**a**) added Gaussian noise, (**b**) added outliers, (**c**) added drift, and (**d**) mixed noise. Plots (**a**) through (**c**) display the mean and 95% confidence intervals, whereas the boxplot (**d**) shows the median and quartiles.

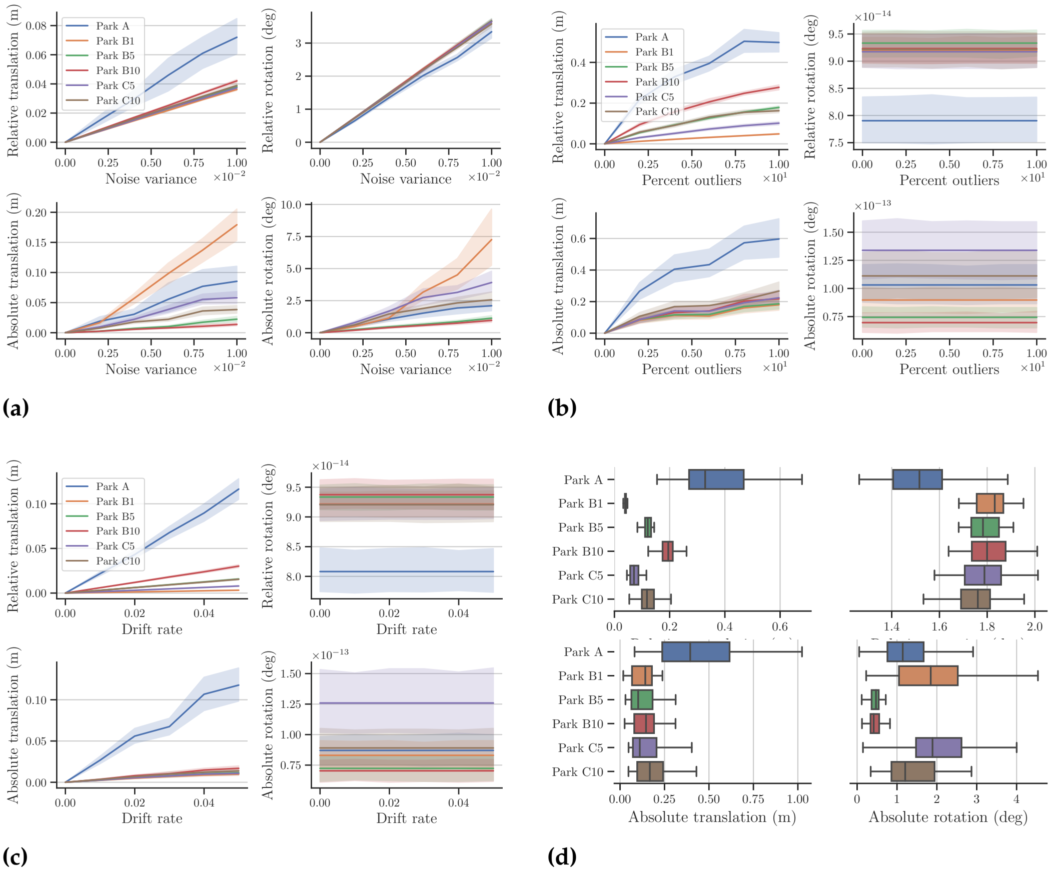

Figure S3. Calibration errors for Park [2] on simulation data for all reference selection methods: (**a**) added Gaussian noise, (**b**) added outliers, (**c**) added drift, and (**d**) mixed noise. Plots (**a**) through (**c**) display the mean and 95% confidence intervals, whereas the boxplot (**d**) shows the median and quartiles.

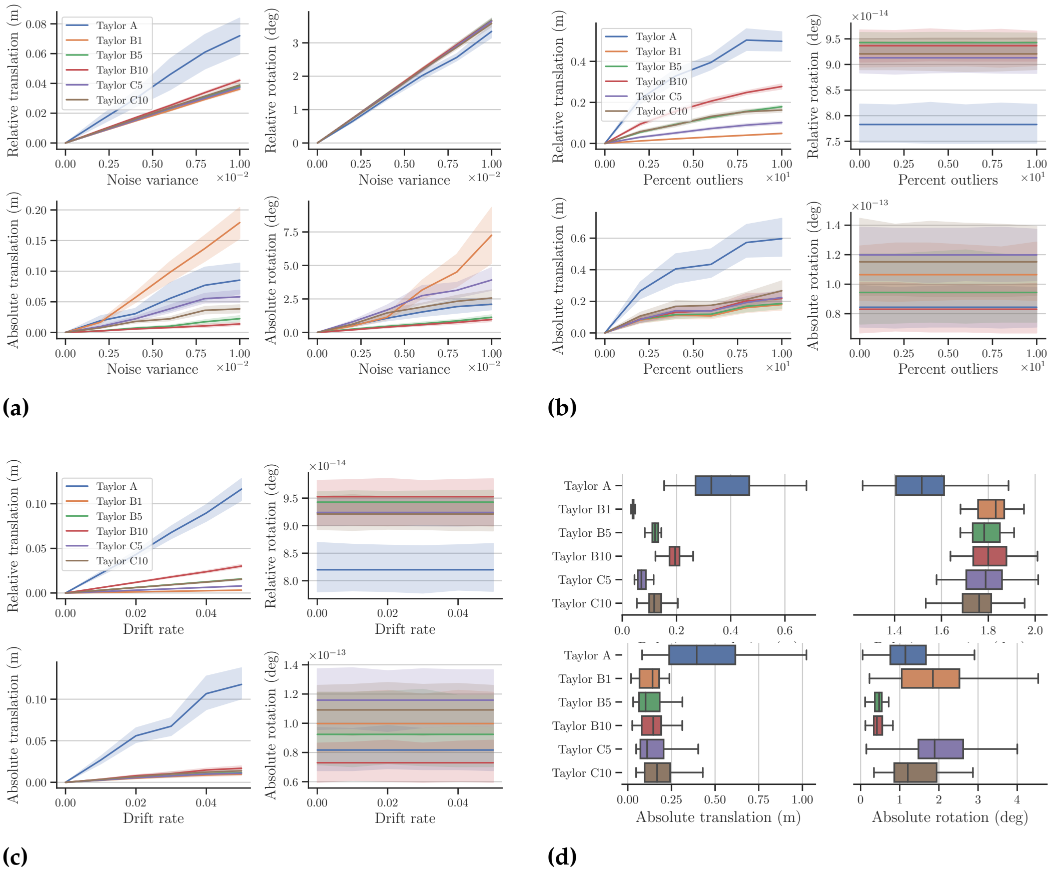

Figure S4. Calibration errors for Taylor [3] on simulation data for all reference selection methods: (**a**) added Gaussian noise, (**b**) added outliers, (**c**) added drift, and (**d**) mixed noise. Plots (**a**) through (**c**) display the mean and 95% confidence intervals, whereas the boxplot (**d**) shows the median and quartiles.

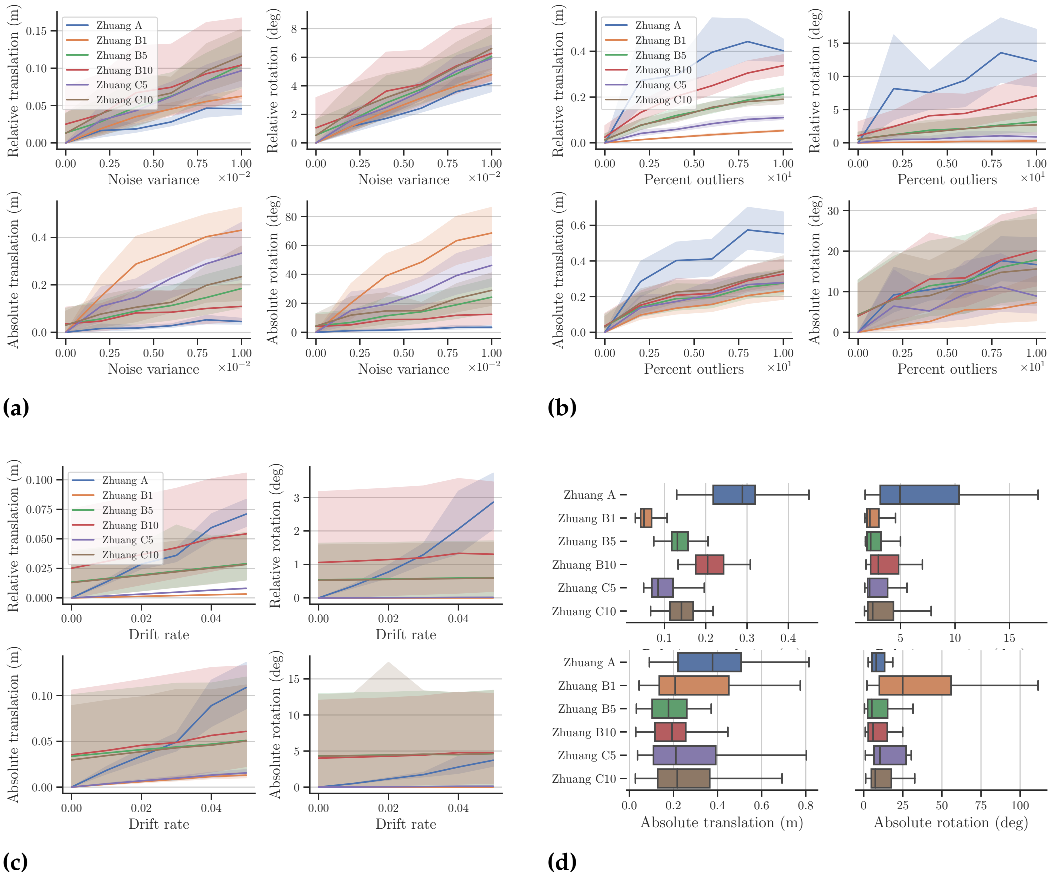

Figure S5. Calibration errors for Zhuang [4] on simulation data for all reference selection methods: (**a**) added Gaussian noise, (**b**) added outliers, (**c**) added drift, and (**d**) mixed noise. Plots (**a**) through (**c**) display the mean and 95% confidence intervals, whereas the boxplot (**d**) shows the median and quartiles.

2. KITTI experiments

Tables S1 to S6 present the camera to LiDAR calibration errors for all tested reference selection methods, whereas Tables S7 to S12 present the camera to camera calibration errors for all tested reference selection methods. Throughout the tables the best values for each column are bolded.

Table S1. Camera to lidar calibration on `2011_09_30_drive_0027` using Ali [1]

	Relative error		Absolute error	
Method	e_{rt} (m)	e_{rR} (deg)	e_{at} (m)	e_{aR} (deg)
Ali A	0.935	2.445	24.598	1.863
Ali B1	**0.042**	**0.111**	0.630	**0.666**
Ali B5	0.170	0.294	**0.342**	0.722
Ali B10	0.298	0.487	0.388	0.778
Ali C5	0.088	0.192	0.749	1.075
Ali C10	0.162	0.273	0.658	0.764

Table S2. Camera to lidar calibration on `2011_09_30_drive_0027` using DNL

Method	Relative error		Absolute error	
	e_{rt} (m)	e_{rR} (deg)	e_{at} (m)	e_{aR} (deg)
DNL A	0.935	2.445	24.598	1.863
DNL B1	**0.042**	**0.111**	0.629	**0.669**
DNL B5	0.170	0.293	**0.342**	0.721
DNL B10	0.298	0.487	0.388	0.777
DNL C5	0.088	0.188	0.750	0.996
DNL C10	0.162	0.273	0.658	0.764

Table S3. Camera to lidar calibration on `2011_09_30_drive_0027` using DNLO

Method	Relative error		Absolute error	
	e_{rt} (m)	e_{rR} (deg)	e_{at} (m)	e_{aR} (deg)
DNLO A	0.926	2.355	8.299	1.755
DNLO B1	**0.038**	**0.112**	0.330	0.441
DNLO B5	0.151	0.290	0.837	0.233
DNLO B10	0.271	0.488	**0.202**	0.232
DNLO C5	0.079	0.187	0.486	0.757
DNLO C10	0.144	0.265	0.300	**0.219**

Table S4. Camera to lidar calibration on `2011_09_30_drive_0027` using Park [2]

Method	Relative error		Absolute error	
	e_{rt} (m)	e_{rR} (deg)	e_{at} (m)	e_{aR} (deg)
Park A	17.068	1.939	30.581	16.190
Park B1	**0.043**	**0.110**	0.618	0.747
Park B5	0.160	0.286	0.368	**0.568**
Park B10	0.325	0.473	**0.183**	0.849
Park C5	0.087	0.165	0.903	0.623
Park C10	0.254	0.260	2.179	1.586

Table S5. Camera to lidar calibration on `2011_09_30_drive_0027` using Taylor [3]

Method	Relative error		Absolute error	
	e_{rt} (m)	e_{rR} (deg)	e_{at} (m)	e_{aR} (deg)
Taylor A	17.068	1.939	30.581	16.190
Taylor B1	**0.043**	**0.110**	0.618	0.747
Taylor B5	0.160	0.286	0.368	**0.568**
Taylor B10	0.325	0.473	**0.183**	0.849
Taylor C5	0.087	0.165	0.903	0.623
Taylor C10	0.254	0.260	2.179	1.586

Table S6. Camera to lidar calibration on `2011_09_30_drive_0027` using Zhuang [4]

Method	Relative error		Absolute error	
	e_{rt} (m)	e_{rR} (deg)	e_{at} (m)	e_{aR} (deg)
Zhuang A	0.973	2.405	23.339	1.789
Zhuang B1	**0.055**	**0.152**	**1.063**	2.899
Zhuang B5	0.187	0.475	2.610	2.357
Zhuang B10	0.323	0.542	3.840	**1.026**
Zhuang C5	0.094	0.279	1.439	2.643
Zhuang C10	0.174	0.340	2.117	1.297

Table S7. Camera to camera calibration on `2011_10_03_drive_0027` using Ali [1]

Method	Relative error		Absolute error	
	e_{rt} (m)	e_{rR} (deg)	e_{at} (m)	e_{aR} (deg)
Ali A	4.668	0.533	43.137	0.366
Ali B1	**0.034**	**0.139**	0.178	0.450
Ali B5	0.155	0.181	**0.074**	0.432
Ali B10	0.305	0.205	0.117	0.435
Ali C5	0.076	0.149	0.513	0.421
Ali C10	0.153	0.169	0.210	**0.342**

Table S8. Camera to camera calibration on `2011_10_03_drive_0027` using DNL

Method	Relative error		Absolute error	
	e_{rt} (m)	e_{rR} (deg)	e_{at} (m)	e_{aR} (deg)
DNL A	4.668	0.533	43.137	0.366
DNL B1	**0.034**	**0.139**	0.178	0.450
DNL B5	0.155	0.181	**0.074**	0.432
DNL B10	0.305	0.205	0.117	0.435
DNL C5	0.076	0.149	0.513	0.416
DNL C10	0.153	0.169	0.210	**0.343**

Table S9. Camera to camera calibration on `2011_10_03_drive_0027` using DNLO

Method	Relative error		Absolute error	
	e_{rt} (m)	e_{rR} (deg)	e_{at} (m)	e_{aR} (deg)
DNLO A	4.882	0.539	41.895	0.454
DNLO B1	**0.034**	**0.139**	0.194	0.416
DNLO B5	0.156	0.182	0.190	0.459
DNLO B10	0.306	0.205	0.381	0.434
DNLO C5	0.076	0.149	**0.159**	**0.345**
DNLO C10	0.152	0.168	0.203	0.407

Table S10. Camera to camera calibration on `2011_10_03_drive_0027` using Park [2]

Method	Relative error		Absolute error	
	e_{rt} (m)	e_{rR} (deg)	e_{at} (m)	e_{aR} (deg)
Park A	11.369	0.497	101.102	3.123
Park B1	**0.034**	**0.139**	0.189	0.425
Park B5	0.157	0.181	**0.078**	0.351
Park B10	0.309	0.204	0.145	0.347
Park C5	0.076	0.149	0.515	0.399
Park C10	0.154	0.168	0.255	**0.342**

Table S11. Camera to camera calibration on `2011_10_03_drive_0027` using Taylor [3]

Method	Relative error		Absolute error	
	e_{rt} (m)	e_{rR} (deg)	e_{at} (m)	e_{aR} (deg)
Taylor A	11.369	0.497	101.102	3.123
Taylor B1	**0.034**	**0.139**	0.189	0.425
Taylor B5	0.157	0.181	**0.078**	0.351
Taylor B10	0.309	0.204	0.145	0.347
Taylor C5	0.076	0.149	0.515	0.399
Taylor C10	0.154	0.168	0.255	**0.342**

Table S12. Camera to camera calibration on `2011_10_03_drive_0027` using Zhuang [4]

Method	Relative error e_{rt} (m)	Relative error e_{rR} (deg)	Absolute error e_{at} (m)	Absolute error e_{aR} (deg)
Zhuang A	4.668	0.533	43.137	0.366
Zhuang B1	**0.034**	**0.139**	0.180	**0.319**
Zhuang B5	0.155	0.181	**0.074**	0.400
Zhuang B10	0.305	0.205	0.117	0.430
Zhuang C5	0.076	0.149	0.513	0.367
Zhuang C10	0.153	0.169	0.210	0.329

References

1. Ali, I.; Suominen, O.; Gotchev, A.; Morales, E.R. Methods for Simultaneous Robot-World-Hand-Eye Calibration: A Comparative Study. *Sensors* **2019**, *19*, 2837. https://doi.org/10.3390/s19122837.
2. Park, C.; Moghadam, P.; Kim, S.; Sridharan, S.; Fookes, C. Spatiotemporal Camera-LiDAR Calibration: A Targetless and Structureless Approach. *IEEE Robotics and Automation Letters* **2020**, *5*, 1556–1563. https://doi.org/10.1109/LRA.2020.2969164.
3. Taylor, Z.; Nieto, J. Motion-based calibration of multimodal sensor arrays. In Proceedings of the 2015 IEEE International Conference on Robotics and Automation (ICRA), 2015, pp. 4843–4850. https://doi.org/10.1109/ICRA.2015.7139872.
4. Zhuang, H.; Qu, Z. A new identification Jacobian for robotic hand/eye calibration. *IEEE Transactions on Systems, Man, and Cybernetics* **1994**, *24*, 1284–1287. https://doi.org/10.1109/21.299711.
